# What happens when a whole-school health promotion research trial ends? a case study of the SEHER program in India

**DOI:** 10.3389/fpsyt.2023.1112710

**Published:** 2023-06-23

**Authors:** Sachin Shinde, Monika Raniti, Amit Sharma, Susan M. Sawyer

**Affiliations:** ^1^Department of Global Health and Population, Harvard T.H. Chan School of Public Health, Boston, MA, United States; ^2^Center for Inquiry Into Mental Health, Pune, India; ^3^Centre for Adolescent Health, Murdoch Children’s Research Institute and Royal Children’s Hospital, Melbourne, VIC, Australia; ^4^Department of Paediatrics, Melbourne Medical School, University of Melbourne, Melbourne, VIC, Australia; ^5^Corstone India Foundation, Patna, India

**Keywords:** whole-school intervention, mental health, adolescents, sustainability, schools, education

## Abstract

**Background:**

Health promotion interventions that are developed and evaluated by researchers and other external providers are at risk of not being sustained beyond the initial implementation period. When delivered by a lay school health worker, the SEHER study of a whole-school health promotion intervention in Bihar, India was found to be feasible, acceptable and effective in improving school climate and student health behaviors. The objective of this case study is to describe the decision-making processes, barriers, and enablers to continuing the SEHER intervention following its official closure.

**Methods:**

For this exploratory qualitative case study, data were collected from four government-run secondary schools, two of which continued SEHER and two of which discontinued it after official closure. Thirteen school staff were interviewed, and 100 girls and boys (aged 15–18  years old) participated in eight focus groups discussing their experiences of the process of continuing the intervention (or discontinuing) following its official closure. Thematic analysis was conducted in NVivo 12 using grounded theory.

**Results:**

No school sustained the intervention as originally delivered in the research trial. In two schools, the intervention was adapted by selecting sustainable components, whereas in two others it was discontinued altogether. We identified four interrelated themes that explained the complex decision-making process, barriers, and enablers related to program continuation: (1) understanding of the intervention philosophy among school staff; (2) school capabilities to continue with intervention activities; (3) school attitudes and motivation about implementing the intervention, and; (4) the education policy environment and governance structures. Suggestions for overcoming barriers included adequate resource allocation; training, supervision, and support from external providers and the Ministry of Education; and formal government approval to continue the intervention.

**Conclusion:**

Sustaining this whole-school health promotion intervention in low-resource school settings in India depended on individual, school and government factors as well as external support. These findings suggest that health interventions will not necessarily become embedded in a school’s operations merely because they are designed as a whole-school approach or because they are effective. Research should identify the resources and processes required to balance planning for future sustainability while awaiting trial results about an intervention’s effectiveness.

## Introduction

In recent decades, there has been growing interest in developing whole-school health promotion interventions by governments, external agencies, researchers and schools (1–3). A whole-school approach defines the entire school community as the unit of change and consists of an integrated set of planned, sequential, school-affiliated strategies, activities, and services intended to promote the optimal development of students on a physical, emotional, social, and educational level (2, 3). These whole-school approaches recognize schools as learning environments that also support health and well-being through health promoting school policies and governance structures, the curriculum, the physical and social–emotional environment, links to families and the wider school community, and school-based health services (2, 3). Whole-school health promotion interventions have been shown to improve a range of behaviors and health outcomes including increasing fruit and vegetable intake and physical activity, reducing tobacco use and bullying (4, 5), and improving social, emotional, and behavioral adjustment (6). A key challenge for schools and education systems is ensuring that effective interventions continue to be sustained after their initial development and evaluation (7). Without this, the investment of time and resources during the start-up phase of research risks being wasted (8).

Although effective whole-school health promotion interventions are implemented in low- and middle-income countries (LMICs) (9), little data are available on the sustainability of these interventions once initial funding or support ceases (3). Definitions of “sustainability” vary, but it can be broadly defined as “the implementation of an effective initiative over a context-dependent timeframe leading to irreversible desirable system change” (10). In high-income countries (HICs), recent evidence shows that school capacity (e.g., resources, leadership), staff motivation and commitment, and the wider policy context influence the continuation of school-based health interventions (11, 12). Also of importance is the ability of schools to adapt and embed those elements of an intervention that are fit for purpose within an individual school, but which are not necessarily consistent with the fixed elements or parameters of the original intervention (e.g., the requirements of a randomized controlled trial) (11, 13). Contextual factors such as the degree of resourcing (e.g., staffing, access to funding), the nature of government involvement, and discrepancies between urban and rural schools are known to shape the success of initial intervention implementation. Not only can these contextual factors vary widely between HICs and LMICs but they are also likely to shape the sustainability of interventions in schools (3, 14). Thus, the most critical factors that sustain whole-school health promotion interventions in HICs may not necessarily be applicable in LMICs or may operate in different ways.

In LMICs in particular, lack of funding, skills and prioritization by governments and schools can result in external providers such as NGOs, private businesses, or research teams initiating and delivering many health promotion interventions, rather than government or school staff (13). Although schools offer a suitable platform for the delivery of specific programs (e.g., supporting girls’ development, and improving nutrition), external partners may have less opportunity than education ministries to align and integrate an intervention to the wider activities within a school. Further, without an explicit focus on skill transfer from external providers to school staff, the sustainability of any intervention beyond the period of program funding is arguably even more challenging in LMICs than HICs, given larger class sizes and fewer opportunities for professional development (15). While these high-level factors have been reported across various LMICs, the complexity and heterogeneity between and within specific countries also need to be considered (2–4).

To this end, we sought to explore the experiences of schools following the implementation and evaluation of one of the largest whole-school health promotion intervention trials ever conducted in India, known as the SEHER (Strengthening Evidence base on scHool-based intErventions for pRomoting adolescent health) study (16). It was anticipated by those who developed and funded the intervention that following the cessation of the externally funded, four-year research project, interested schools may well be able to sustain the SEHER intervention. This was considered likely due to the trial’s positive effects on school climate and student health and health behaviors such as less bullying and fewer depressive symptoms, and because whole-school principles underpinned the development of the intervention which aimed to align and embed various health promotion actions into daily school practices (17). Further support for this perception came from the results of the pilot study and first follow-up assessment (8-month follow-up after baseline, Box 1), which showed improved school climate and adolescent health outcomes in the SEHER Mitra-led arm schools when compared with the schools in control and teacher-led intervention arms (16, 17). The implementation of the SEHER intervention mirrored the reality of school health programs delivered in LMICs where there is typically high reliance on an external funder and implementing body (19). We saw this as a unique opportunity to shed light on the sustainability of the different elements of the SEHER intervention following the end of the trial.

**Box 1: Overview of the SEHER trial (16, 17).**

**Aim**: To evaluate the effectiveness and cost-effectiveness of SEHER (**S**trengthening **E**vidence base on sc**H**ool-based int**E**rventions for p**R**omoting adolescent health program), a whole-school multi-component health promotion intervention led by lay counselors or teachers (i.e., SEHER “Mitra” meaning *friend*) in government-run secondary schools to promote school climate and thereby improve adolescent health outcomes.

**Study setting and duration**: 74 government-run secondary schools in the Nalanda district of Bihar, India, a poor district which has an adult literacy rate (64.4%), well below the national average (77.7%). Bihar is the third most populous state in India, home to more than 103 million people, of whom 22.5% are aged 10–19 years. Education is provided primarily by the state government’s Department of Education (18).

There were 25 schools in the SEHER Mitra (SM) arm, 24 schools in the teacher as SEHER Mitra (Teacher SM) arm, and 25 schools in the control arm.

**Design**: Cluster randomized controlled trial using repeated cross-sectional surveys (April 2015–February 2017). Three assessment points were at baseline (June 2015), 8 months after baseline (March 2016), and 17  months after baseline (December 2016).

**Inclusion criteria**: All students in grade 9 (13–15 years) and present on the day of assessment were eligible to participate in the study.

**Total study participants**: 13,035 at the baseline, 14,414 at the 8-month follow-up, and 15,232 at the 17-month follow-up.

**Intervention**: SEHER, a multi-component whole-school intervention was designed within the Health Promoting Schools (HPS) framework. The intervention’s conceptual framework emphasizes the importance of a positive school climate (i.e., supportive relationships between school community members, a sense of belonging to the school, a participative school environment, and student commitment to academic values).

The intervention identified four priority areas for action: promoting social skills among adolescents; engaging the school community in school-level decision-making processes; providing access to factual knowledge about health and risk behaviors to the school community; and enhancing problem-solving skills among adolescents.

The intervention strategies were organized at three levels: whole school, group, and individual. Whole-school level activities included establishing a School Health Promotion Committee; conducting regular awareness generation activities during a school assembly; organizing competitions; providing a platform for students to raise their concerns, complaints, and suggestions anonymously through a suggestion box; running a monthly wall-magazine; and, developing and implementing healthy school policies. Group-level activities included: forming and running classroom-based peer groups to address students’ concerns; a workshop for students on effective learning techniques; and a workshop for teachers on effective disciplinary practices in the school. Individual-level components included providing counseling services to students who self-referred or were referred by teachers for health complaints, social and emotional problems, and academic difficulties. This intervention was delivered either through a trained lay counselor, SEHER Mitra (SM, “friend”), or a trained teacher, called a teacher SEHER Mitra (Teacher SM).

**Selection and training of SM/Teacher SM**: The SMs were members of the local community who were over 18 years old, had completed at least a bachelor’s degree, and were fluent in the local language (Hindi). The Teacher SMs were nominated by the school principals, had a minimum of 5 years of teaching experience in secondary schools, had 15 or more years of service remaining, and did not teach the Adolescent Education Program curriculum (control intervention). The SMs and Teacher SMs underwent a week-long separate training, with an identical curriculum. This was followed by in-service training through separate monthly group meetings for SMs and Teacher SMs. Eight supervisors provided support and supervision to a combination of SMs and Teacher SMs through three planned visits per month.

**Comparison intervention**: The Bihar state government-run Adolescent Education Program (Tarang) was delivered in all three arms of the study. A trained teacher from each school ran classroom-based sessions on the process of growing up, establishing positive and responsible relationships, gender and sexuality, prevention of HIV and other sexually transmitted infections, and substance use. These topics were delivered during 16 h of sessions each academic year.

**Primary outcome**: School climate measured through the 28-item Beyond Blue School Climate Questionnaire.

**Secondary outcomes**: Depressive symptoms, frequency of bullying, attitude toward gender norms, knowledge of reproductive and sexual health, and violence (perpetration and victimization).

**Main findings:** Compared to the control group, the lay counselor-delivered intervention improved school climate, depression, bullying, attitude toward gender equity, violence victimization, and violence perpetration. These outcomes had larger effect sizes at the end of the 2nd follow-up than they did at the 1st follow-up. No intervention effect was found at either follow-up point for the teacher-delivered intervention. SM-led interventions cost US$3213 per school ($15.0 per student) and $1,390 per school ($7.4 per student) more than the existing Adolescent Education Program.

The objectives of this study were to describe the decision-making processes, barriers, and enablers around the continuation of the SEHER intervention in schools in Bihar, India following its official closure. Our wider goals were to consider the ways in which these barriers could be overcome.

## Methods

### Context

The SEHER trial took place in the Nalanda district of Bihar, India between June 2015 and January 2017. Box 1 provides details of the four-year research study, which was conducted by Sangath, a not-for-profit organization in India. In short, the large randomized controlled trial took place in 74 of the 141 schools in the district, out of which one-third were allocated to the intervention group delivered by a lay counselor, named SEHER Mitra (SM; “Mitra” meaning “friend” in the Hindi language), one third to the intervention group delivered by a teacher, named Teacher-SEHER Mitra (Teacher SM), and the rest were assigned to a control group. The control group intervention was delivered in all three arms and consisted of a government-run Adolescent Education Program (AEP) delivered by a trained teacher. The SEHER intervention was designed and implemented in collaboration with the Department of Education, Government of Bihar. Following the final assessment of students in February 2017, Sangath formally closed the program at a joint meeting of all participating school principals, and program staff.

### Study design and school sample

This study was an exploratory qualitative case study of four SEHER trial schools: one school from each of the two active trial arms where the intervention activities were continued and one school from each of the two trial arms where intervention activities were discontinued after the official closure of the trial. The case study method was chosen as it permits flexibility to explore program evaluation and development and testing of theory, as well as describing and interpreting research findings within the unit of analysis (20). The unit of analysis for this study was the continued and discontinued schools. Following the closure of the program, two schools intended to continue the intervention, one from each of the two active intervention arms of the study. Both these schools were included. Schools that discontinued the intervention were purposefully selected based on their willingness to participate in the study and their geographic proximity to the schools that continued.

### Data collection

The study was conducted between September and December 2020 in the middle of the school year, which ran from April 2020 to March 2021 in Bihar. We conducted face-to-face semi-structured individual interviews with school staff and focus group discussions (FGDs) with randomly selected students in grades 9 and 10. In each selected school, the principal, the SM/Teacher SM (only in continued schools), the AEP teacher, and one purposively selected teacher, based on availability, were interviewed. Two FGDs were conducted within each school (one each with boys and girls in the co-educational schools). Schools were closed between March and August 2020 due to the COVID-19 pandemic; data collection took place when schools were formally open. For each school visit, researchers followed local government and school infection control procedures which included physical distancing, mask-wearing, and hand hygiene. The details of the sample are shown in Table 1.

**Table 1:** Details of interviews and focus group discussions by case study schools.

	Intervention continued *n* = 2 schools	Intervention not continued *n* = 2 schools
**Type of schools**		
Co-education school	1	2
Only girls’ school	1	–
**Intervention led by**		
SEHER-Mitra led school	1	1
Teacher as SEHER-Mitra school	1	1
**Number of focus group discussions**		
Male students from grades 9 and 10	2 (23 boys)	2 (25 boys)
Female students from grades 9 and 10	2 (27 girls)	2 (25 girls)
**Number of interviews**		
School principal	2 (both males)	1 (1 female)
Teacher-as SEHER Mitra	1 (male)	1 (female)
SEHER Mitra	1 (male)	–
Adolescent Education Program teacher	2 (both males)	2 (1 female and 1 male)
Fellow teacher	1 (female)	1 (male)

The staff interviews and FGD guides (see Box 2 for examples) covered the following topics: socio-cultural context and school governance structures; understanding of the intervention and its impact; roles of various school community members in the intervention activities; decision-making processes for continuing or discontinuing the intervention activities; and enablers and barriers considered in making the decision. In the continued schools, we also explored the current challenges, enablers, and opportunities in continuing with the intervention activities and possible ways to address the challenges. All interviews and FGDs were conducted in Hindi, the local language, by trained interviewers who were familiar with the SEHER intervention principles. Interviews lasted between 30 and 45 min, and FGDs lasted between 45 and 60 min. Each interview and FGD was audiotaped, transcribed verbatim, and translated into English. The interviewers’ notes were added to the transcripts.

**Box 2: Example questions for school staff interviews and student focus group discussion.**

**Example staff questions:**

- What were the reasons for the continuation/discontinuation of the SEHER intervention in your school after the official closure by the implementing organization?
- How did you decide on continuation/discontinuation?
- Who was involved in making this decision?
- What role did the principal play in this decision-making?
- Were the students involved in this process? If so, how?
- What kind of help did you receive from Sangath to continue the SEHER intervention in your school?
- What kind of help did you receive from the Department of Education to continue the SEHER intervention in your school?
- What factors posed a challenge to continuation? How did you overcome these challenges?

**Example of student focus group vignette and questions:**

Case vignette: Aman is a 16-year-old boy studying in grade 9. Aman belongs to a poor family. His parents are working as farm laborers. For the last couple of months, they have been asking Aman to stop going to school and help them with their work. Aman wants to study but also cannot say no to his parents. Aman is constantly worrying about this situation and does not know what to do.

- How likely do you think that could happen to anyone in your school?
- If Aman was your classmate, what would happen to him?
- Who could help Aman at your school? How?
- What activities are conducted in your school to help students like Aman?
- Who conducts these activities? How often do they occur?
- What topics or issues are discussed during these activities?

### Analyses

We used a grounded theory approach to thematic analysis (21–25). By using this approach, both deductive and inductive processes of qualitative data analysis could be combined. The themes covered in the interview and group discussion guides constituted an *a-priori* framework (i.e., deductive) for analysis, while the perceptions and views expressed by participants allowed the identification and progressive refinement of critical themes that were grounded within the data (i.e., inductive). The following steps were taken for the data analysis and interpretation. In the first stage, two authors (MR and SS) read and familiarized themselves with the data. In the second stage, these two authors selected a mix of interviews and group discussions across participant categories and applied open codes through categorizing parcels of data (25). Then, they reviewed the coded transcripts and based on the codes and original research questions, defined and collated codes into potential themes to develop a codebook. In the fourth stage, they independently applied the codebook to five randomly selected transcripts (25). Post-coding, a summative table was prepared to discuss and resolve all instances where consensus was not complete. At this stage, the cycles of inductive elaboration of themes from the data were followed by their deductive application to the data. The procedure ensured that data within each code were coherent and that there were clear distinctions between codes. This process also allowed the existing constructs of sustainability of school-based health interventions to be grouped and compared. The framework was revised in discussion with SMS until the three researchers were satisfied that it fully reflected the data. The revised codebook framework is shown in Supplementary file 1.

Once refined, the codebook was applied to all transcripts, populating a matrix framework with verbatim and summarizing data from the transcripts using NVivo 12 software (26). Ongoing charting of each interview transcript took place during and after this process, comparing new data with earlier transcripts. This ensured that the resulting matrix provided a detailed and accessible overview of the data populating each theme and subtheme from every respondent. The matrix framework enabled exploration of the data by both theme, and respondent-type, which allowed us to develop a detailed description of each theme and subtheme, and to detect patterns and associations between and across themes in the data (25).

### Ethical considerations

This study was approved by the Human Ethics Advisory Committee at The University of Melbourne, Australia (Ethics ID 2057035.1). Written informed consent was obtained from all adult participants. For participants under the age of 18, participant assent was obtained before each FGD, using parental opt-out consent due to the low-risk nature of the research.

## Results

### Description of schools

In total, 2,502 students were enrolled in the four schools selected for the case study; three schools had less than 300 students while one school had 1,658 students. Three of the four schools were co-educational (58.8% boys) while the other was an all-girls school. In total, 36 teachers were employed across the four schools (Mean 10.5 ± 4.12; range: 6 to 16), with an additional 18 vacant teacher positions (Mean 4.5 ± 3.0; Range: 2–8).

### Description of participants

Thirteen school staff were interviewed including four principals, seven teachers (including three AEP teachers), one SM, and one Teacher SM. Eight FGDs (5 with girls, 3 with boys) were conducted with 100 students (58 girls, 42 boys; aged 15–18) to discuss their experience of the intervention after its official closure.

### Educational context

Both students and teachers described Bihar’s socio-cultural milieu and the relatively poor state of its education system. Teachers pointed out inadequate school infrastructure, including inadequate drinking water and sanitation facilities, insufficient and unsafe classrooms, understaffing in schools, delays in school infrastructure development activities due to bureaucratic procedures, and poor monitoring by government authorities.

Among the major barriers to completing 12 years of schooling, students pointed to poverty, family reluctance to educate girls in comparison to boys, gender discrimination at home, teacher absenteeism, and a poor sense of connection to the school. During the group discussion, a female student expressed:

“Students like us, living in villages, face multiple problems. Students drop out due to poor financial circumstances. Girls drop out of school because they are expected to assist in household chores and domestic work, while boys are expected to earn money to support the family. Boys are sent to work in factories, shops, and hotels in big cities. Many girls are forced to get married before they finish their education or asked to sit at home to support the education of their brothers. Many do not attend school during 'those' days [referring to menstruation] since there is no separate toilet for girls.”

School staff shared these understandings, with one of the interviewed teachers saying:

“Most of the students in our school are from lower castes and poor families. They are the first learning generation in their family. They [their families] do not understand the value of education and are indifferent to the education of their children, especially their girls, who they do not want to educate and [who] marry before they turn 18.”

Students and teachers noted the lack of physical infrastructure and resources in the schools, including insufficient classrooms and seating arrangements, the dilapidated condition of school buildings, and the lack of safe drinking water, and toilet and sanitation facilities. Teachers also commented on the lack of human resources, with one saying,

“All of the teachers are overburdened with academic and non-academic responsibilities. Our school has eight vacant teaching positions, and this is more or less the case throughout the entire state. Our school has about 80 students in each classroom…very difficult for a single teacher to manage crowded classrooms.”

### Decision-making processes, barriers, and enablers to continuing the intervention after its official closure

**Figure 1:**
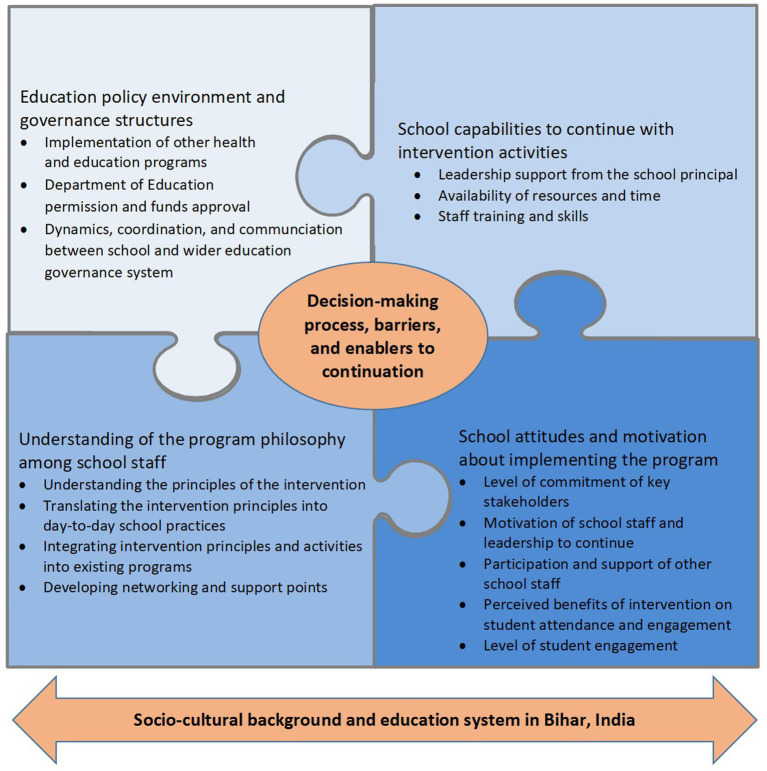
Identified themes around the decision-making process, barriers, and enablers to implementation that led to continuation or discontinuation of the SEHER intervention in schools.

No school sustained the intervention as originally delivered during the trial. Two schools ceased the intervention altogether and two schools adapted the intervention by selecting components that were perceived to be sustainable (e.g., no or low cost, could be incorporated within the existing school schedule). We identified four major themes related to the decision-making processes and implementation factors of whether a school continued or discontinued the intervention activities after its official closure (see Figure 1).

#### Understanding of the intervention philosophy among school staff

The degree to which school principals and teachers understood the SEHER intervention philosophy as a whole-school approach that had benefits for health as well as learning impacted its continuation. Teachers who were able to translate and generalize the SEHER principles into day-to-day activities of the whole school, as well as classroom-level pedagogy, were more likely to adapt the intervention, even under low resource conditions. One school, whose SM continued with intervention activities, adjusted internal resources to pay for the SM’s salary and continued with the no-cost and easy-to-implement intervention activities in order to sustain the engagement of students. The majority of these activities took place at the school and group level, including awareness generation activities during the assembly sessions, weekly classroom sessions, running a monthly wall-magazine and competitions, occasional workshops for students and teachers, and regular peer group activities. The principal of this school mentioned,

“Our team continued with the activities that were easy to implement and did not require additional resources. We reuse the wall magazines designed when the program was supported by Sangath. During the general assembly, we still discuss various topics, conduct yoga sessions, and occasionally present skits about health issues. Moreover, [SEHER Mitra] organizes competitions for students, but we have reduced the number of competitions and do not distribute prizes. Instead, we commend the winners during the assembly. Our students are still encouraged to submit problems and concerns through the speak-out box so that [SEHER Mitra] can get in touch with them. This program aims to boost students' participation in school and classroom activities…teaching staff who have understood this principle encourages students to participate through group activities.”

There was, however, a general lack of understanding among teachers and students about the principles of the intervention (Box 1). For example, there was no mention among the principals and teachers interviewed that the main goal of the intervention was to improve the physical, social, and emotional climate of the school. Among the four priority areas identified by the SEHER intervention for action (see Box 1), the principals and teachers only mentioned two as priorities, namely providing students with factual information about health risk behaviors, and helping them resolve personal problems. In the interview with AEP teachers, they noted some overlap in the content between SEHER and AEP but differences in delivery methods. One of the male AEP teachers from the intervention continued school reported,

“SEHER and Tarang (Adolescent Education Program) both instruct students about risk behaviors such as tobacco chewing and smoking, unprotected sexual practices, physical inactivity, and violence. I teach these issues in the classroom and the SEHER teaches these through an activity-based approach. One of our staff members is also trained to be a counselor for the students, so that teacher helps girls deal with their problems; when they have health concerns, they also tell me about them. Since an organization closed the SEHER program, the SEHER teacher and I work together to benefit students.”

When asked about the SEHER intervention’s objectives, focus group participants described some of the activities, such as wall magazines, assembly sessions, and the speak-out box in detail. One or two students in each FGD reported that the SEHER intervention’s primary objective is to help students solve personal and school-related problems. Notwithstanding this, they also described many instances in which school personnel did not respond to their concerns. A group of girls from one of the continued schools shared,

“Sometimes no matter how many times we write about a problem, it is not solved. Nobody takes any action related to our problem… We wrote about the students’ toilet. It is mostly locked and when it is opened it is not clean… We write about it but no action is taken.”

Interviewer: *Did you discuss it with the teacher?*

“Yes. We have told him about it…He said he would discuss it with the Principal…No action is taken so far.”

The SEHER intervention included a number of overarching components which were intended to support a positive school climate, including a school health promotion committee, health policies, and the involvement of parents (Box 1). Schools did not sustain these elements. Many teachers saw the opportunity to merge SEHER activities with those of other programs due to similarities in content or structure and had done some of this in the schools where the intervention activities continued. However, several challenges were identified around doing this, including the unwillingness of other program staff to be flexible in this way, which was compounded by a lack of time and other resource constraints, as well as a lack of endorsement from the Department of Education (See Policy environment and governance structure below). As one teacher from the intervention discontinued school explained,

“When Sangath was implementing the program, it was going on very well. Since the organization has left, there is no one to support and look after the program in our school… the program is not implemented anymore. Earlier a SEHER team member would visit our school once a month and plan activities with the [SEHER Mitra], and all teachers were informed about the activities in a monthly meeting. All of this is stopped now. The main reason is we do not have any resources to implement the program activities.”

#### School capabilities to continue with intervention activities

The capabilities of schools to continue with the intervention emerged as a strong theme. This included: the need for strong leadership; administrative and management support from the principal; the willingness of the teacher SM to continue with the intervention activities; the need for ongoing training and skill development for teachers to be able to continue the intervention activities; the need for dedicated time and resources (human, financial, technical and material); the need for data (e.g., around intervention benefits) to inform decision-making; and the presence of support networks between teachers within the school and between staff across other programs.

Conflicts of interest, leadership disputes, and disagreements among teachers characterized the decision-making process in schools that discontinued the program. In schools where some intervention activities were continued, teachers lauded the role of the principal as a leader and facilitator in creating a consensual plan of action to resolve conflicts and provide strategic guidance.

A few teachers mentioned that it was important to protect the process of decision-making from misunderstandings, conflicts, and interpersonal dynamics, and to share responsibilities based on collective interests to assure the long-term running of the intervention. Teachers mentioned that the decision-making process involved discussing clear roles for implementing intervention activities. For example, as one teacher from the discontinued schools explained,

“Teachers who are involved in the program activities should be exempted from other responsibilities from the school routine so that they can devote that time to implement program activities…the SEHER program activities require additional time and effort from the teachers. If exempted from other responsibilities, teachers can devote some time to plan and execute program activities on a regular basis and also think about and address the issues faced by the students. At the same time, other teachers need to support in implementing the program activities…we could not reach a consensus on this…the output was obvious.”

In schools where some intervention activities were continued, principals and intervention facilitators noted that they used the materials provided by Sangath and developed by students from earlier years such as wall magazines, posters, charts, and competitions. The intervention implementers also said that the initial training they received was beneficial. One of the intervention facilitators shared,

“Sangath trained us extremely well. We had very intensive weeklong training at the beginning of the program followed by monthly training sessions. Moreover, we were provided with written guidelines and supporting materials that enabled me to continue with activities such as general assembly sessions, the speak-out box, and the monthly wall magazines. I repurposed the wall magazines that we previously created due to limited resources. However, we would benefit from more resources.”

Yet in all schools, it was clear that should the intervention facilitator (the lay counselor or teacher) who is currently employed at the school and originally trained by Sangath leave the school, the intervention would be discontinued because fellow teachers did not have the training nor resources to continue the intervention activities. This was a key consideration in the decision-making process. One teacher from a discontinued school stressed the need to build skills and knowledge across teachers in the school,

“Other teachers should be trained to implement program activities… When all the teachers are trained in implementing the program activities then the program activities can be distributed among teachers and one teacher will not be overburdened with the delivery of the program activities.”

#### School attitudes and motivation about implementing the intervention

Teachers and students generally had positive opinions about the benefits of the intervention for students. They believed that the anticipated benefits for students (e.g., the program created conditions conducive to learning such as better attendance, better involvement of students in the classroom, and general improvement in interactions between students and teachers) was a factor that contributed to the decision to continue the intervention. Principals and teachers noted that economic and other hardships in the community and society affected students, and that they viewed the intervention as a way for schools to help students address personal problems and provide them with the necessary skills to be prepared for the future. One teacher from a continued school noted benefits to the socio-emotional environment of the school,

“Students in the rural area are naturally shy. This program encouraged them to be vocal about their needs… either through raising them in group meetings or the classroom or through a written chit dropped in the speak-out box… students became vocal. They would ask questions during the classroom sessions. They would discuss topics like child marriage, the dowry system, the education of girls, depression, and so on, in debates. There were activities on mental health… how to handle stress, how to manage anger, and relationships. This all helped the students.”

Another teacher from a continued school emphasized the benefits to student-teacher relationships,

“This program brought some sort of schedule and discipline among the students. We have seen improvement in the student-teacher relationship… Students started sharing a bond with the school and the teachers… the girls could approach me and other teachers with their problems. This is important to improve their health and overall life.”

Others mentioned improvements in school attendance and student engagement in learning. One noted “*We did observe an increase in students’ attendance during the SEHER program”* while another said, *“It helps in improving students’ engagement.*”

In the FGD, many students appreciated the information they received on a range of topics through SEHER activities and noted that these would otherwise not be discussed in school or in their communities. Additionally, they valued the opportunity to engage with fellow students and the intervention facilitator in fun activities that were not part of their regular studies but that helped them to gain knowledge and skills. Several students shared anecdotes about the facilitator helping them resolve personal problems while ensuring confidentiality. One of the girls from the intervention continued school shared,

“I was going through a personal problem. My parents wanted to stop my education and get me married. I was disturbed due to this fact and could not concentrate on anything. I did not know what to do so I went to SM sir. He patiently listened to my problem and assured me that nothing of that sort would happen. He asked me whether I would be okay if the principal talked to my parents, which I thought was an okay thing to do. My parents were called to the school. Our principal and SM sir discussed this issue with my father a couple of times and my parents agreed to continue my education and not to think about my marriage before I complete grade 12 education”.

Despite this, in all four schools, staff had mixed opinions about whether the intervention should be continued or not. Discontinuation was more likely when the principal did not drive decision-making, when teachers were unwilling to take on additional responsibilities, and when teachers had little interest or motivation to continue, which was influenced by ineffective or even conflictual interpersonal dynamics between teachers. In contrast, in all schools, relationships built on trust, authenticity, and cooperation were described as being important for the intervention to continue. As one teacher from the intervention continued school shared,

“[Teacher (SEHER Mitra)] and [teacher (Tarang)] work collaboratively, and the rest of the teachers in our school support them. Our school's principal is also cooperative and supports teachers in executing various activities…we work as a team.”

Thus, the level of commitment and dedication of key stakeholders (i.e., principal and SM/Teacher SM) and clear understanding, support, and motivation from all stakeholders about what was needed to continue the program were key considerations during the decision-making process.

Teacher attitudes extended beyond the specific aspects of the intervention to encompass their overarching values about teaching and learning, including the role of education in society. One teacher highlighted that the sustainability of the intervention partially lay in the school staff appreciating that school is not just a place for education, but also a place for building a foundation for a healthy and productive life,

“This decision [to continue the program] depends on what is the attitude and opinions of the teachers towards the program… Teachers in this school believe that the school is the place where students can be helped with their problems and [that it] influences the students’ future life. That is why we have continued [the program].”

Engagement with students in decision-making around plans for the intervention was apparent in schools that continued with the intervention, even if in a very perfunctory manner around sharing with students that the intervention was officially coming to an end. However, some teachers noted the extent to which students had been asked for their opinions about the intervention’s closure and suggested that this had contributed to the school’s decision-making process. One of the principals from a continued school shared,

“We informed the students of the closure of the program when the organization [Sangath] announced it. [Teacher] asked the girls how they felt about continuing the program activities in each classroom… that batch of girls is no longer in the school, (but) their opinions were important to the continuation of the program activities for upcoming batches.” (Principal of a continued school)

In contrast, students were not visible in decision-making in any way in the two discontinued schools. A teacher from a discontinued school mentioned,

“There was no need to consult with students. There are a few hundred students at the school, and we know that they may have different opinions. Some of the students kept asking why we weren't conducting the program activities. We told them that the organization had closed.” (Teacher from a discontinued school)

#### Education policy environment and governance structures

Teachers cited several barriers to intervention continuation that reflected aspects of policy and governance. These included the challenges of the top-down or vertical implementation of health and education programs in the state of Bihar; the challenge of “red tape” about complex bureaucratic processes around approval for funding; hierarchies and dynamics within the school and education system; the fragmented delivery of multiple health programs in schools; and lack of coordination between the Department of Education and the implementing agency (Sangath).

No school reported communicating with the Department of Education following the official cessation of the intervention, yet the need for a government directive to continue the intervention was frequently raised as a critical enabler of its continuation (See Overcoming barriers to continuation below). As one teacher from a discontinued school described,

“We cannot go against the Government's directives…We are ready to spend the school development fund for these programs but a proper directive should come from the Government. Right now, there is no such guideline…”

Another teacher from a continued school highlighted the need for government investment in teacher training and school capacity building,

“The Department should acknowledge the program and its importance. The Department can train the teachers and principals… give some freedom to the school principals to utilize the school funds.”

The uniqueness of each school and situation presented many challenges for the sustainability of the intervention after official closure and for the implementation process in the present - and future. Three common themes emerged as affecting sustainability at the school level: lack of a strategic plan by the organization to hand over the intervention to the Department of Education and schools, lack of preparation by the schools to continue implementation without help from the organization, and the schools’ actual implementation without any financial support or technical assistance. According to the interviewed teachers and principals, the organization did not work with the Department of Education to hand over the implementation and governance of the intervention nor did it work with participating schools to develop a roadmap for integrating the intervention activities into the daily school schedule in the absence of any resources. According to one of the principals from a discontinued school,

“In a joint meeting of all the schools, we were informed about the program closure. Because they have been clear about it since the start of the program, I cannot blame them. However, the organization could have collaborated with the Department of Education to continue the program activities with minimal assistance. In each school, more teachers could have been trained so that the schools could continue their implementation. At the end of the program, only one meeting was held to inform teachers that the program has closed… we would have liked the program team to help us continue the program at school.”

### Overcoming barriers to continuation

Overall, participants described several opportunities that could help overcome a number of barriers to continuing the intervention, some of which have been already reported. These include: adequate allocation and accessibility of materials to deliver intervention activities; provision of training, support and supervision from both the Department of Education and Sangath; training of all teachers instead of a single teacher to facilitate team-based program delivery and ensure intervention sustainability through staffing changes; and rewards for teachers to deliver the intervention activities. As previously noted, formal communication from Sangath to the Department of Education was raised by a number of staff as a necessary strategy to help shift intervention responsibility and leadership from Sangath to the Department of Education.

One of the teachers described how government officials must relinquish their bureaucratic role and allow schools to handle funds and other resources. They also described the value of trained teachers sharing their learning with colleagues.

“For a school principal to make a school-level decision, there are a lot of procedural requirements. It is important that the Department of Education allows the school principals to make some decisions on their own regarding the school development funds to build school infrastructure and implement programs like SEHER and Tarang (AEP). At the same time, at the school level, teachers need to function as a unit and share information. When the program was implemented by Sangath, we had no access to the program guidelines and resource materials. If all the teachers had received copies, there would have been an increase in cooperation and engagement on their part.”

Prior to the SEHER program closure, principals and teachers suggested that team training at each school should have taken place to prepare the school as a unit to continue implementing the intervention, rather than burdening one teacher with intervention responsibilities. Beyond this, however, in the continuing schools, the SEHER Mitra also emphasized the need for ongoing coaching and supervision. For example, one noted,

“It is not always easy for me to provide solutions to the issues shared by the students. Previously, when Sangath was implementing the program, I was able to communicate with the supervisor regularly and resolve any issues that I had. Now, I do not know who to contact… the program is discontinued in almost all schools so I cannot contact my peers, nor can I contact anyone in the Department of Education…this is a pressing issue.”

According to one of the principals interviewed,

“To sustain the program, there will be a need for shared energy, commitment, and passion from all levels of leadership… the school principal needs to provide support and supervision to the teachers while the department officials need to provide funding and materials. Sangath should have coordinated with the Department of Education, which it did not. Nevertheless, they can initiate the discussion with the Department to continue program activities with minimal support and supervision… in essence, leadership and dedication at all levels are what is needed to move forward with such a program.”

## Discussion

In setting out to describe the decision-making processes, barriers, and enablers to continuing the SEHER intervention in Bihar, India following the completion of an effectiveness trial, we wished to explore the elements that promote sustainability of effective whole-school health promotion programs as little is known about the sustainability of programs that are developed and evaluated by external providers in LMICs after research funding ceases. We found that none of the four schools in this case study sustained the intervention as originally delivered. Two schools adapted the intervention by selecting components that were perceived as sustainable (e.g., no cost, activities could be incorporated within the existing school schedule), while two schools completely ceased the program. Overall, we identified four interrelated themes related to the sustainability of the intervention: (1) understanding of the *intervention philosophy* among school staff; (2) *school capabilities* to continue with intervention activities; (3) school *attitudes and motivation* about implementing the intervention; and (4) the education *policy environment and governance structures*.

These findings are broadly consistent with existing evidence from HICs. In their seminal review of the sustainability of 18 school-based health promotion interventions in HICs, Herlitz et al. (11) found that no intervention was sustained in its entirety (e.g., some intervention components were continued while others were not). Indeed, overall costs, ease of implementation of intervention activities, and adaptation of the intervention to suit a school’s day-to-day operations were found to be part of the sustainability process (2, 3, 11–13). The importance of school capacity (e.g., resources, leadership support, and trained staff), staff motivation and commitment (e.g., staff confidence, perception of intervention benefit to both health and education, whole-school engagement) were also important and align with the themes we identified (3, 11). While the review by Herlitz et al. (11) identified the role of the wider health policy environment, this case study identified the critical role of the Department of Education in providing the necessary conditions (i.e., endorsement, funding allocation, communication with external providers and schools, leadership, student-teacher ratio) for sustainability to occur. The importance of national and sub-national governance structures, led by the Ministry/Department of Education is increasingly being recognized as critical to the sustainability and scalability of these programs (2, 3).

We found no apparent relationship between those schools in the trial arm where the SEHER intervention was found to be effective (i.e., SM active intervention arm) or ineffective (i.e., Teacher SM active intervention arm) and whether they continued the intervention. This is also consistent with the Herlitz et al. (11) review, which showed that while the effectiveness of a school-based health promotion intervention was not associated with its sustainability, the perception of benefit was. This was also the case in this study, where school staff and students perceived the intervention to be effective and valuable for many outcomes, despite several of these not specifically being measured (e.g., school attendance) and only the SM arm being effective relative to the AEP control arm in the original SEHER trial (16). This perception appeared to drive teacher motivation and commitment to continue with the intervention, even in these low-resource conditions, in which limitations around relying on teacher motivation were also apparent.

The ability of school staff to identify opportunities to integrate intervention activities with existing curricula or other health programs already being delivered was critical to sustainability. Yet, this case study showed that schools “defaulted” to a programmatic approach when deciding whether to continue the intervention. That is, schools perceived the “program” to be the activities and curricula-based elements designed to improve health knowledge (usually about a single problem or health topic), including one-on-one counseling of students, rather than continuing more structural (e.g., school policies, committees), pedagogical (e.g., how teachers teach and engage with students and student learning, rather than what they teach) or environmental elements (e.g., cleanliness of female toilets, access to the library, engaging in respectful conversations, including students in decision-making or listening to their concerns, providing a safe place for students). In continued schools, the more overtly programmatic elements were continued, while in the discontinued schools, these were the same elements that were the focus of discussion when determining whether the intervention could be continued. This is particularly noteworthy given that the SEHER trial was found to be effective in its aims to improve the school climate (i.e., supportive relationships between school community members, a sense of belonging to the school, a participative school environment, and student commitment to academic values) rather than having a more narrow focus on only improving health knowledge or outcomes. This is in marked contrast to the control arm of the trial, the AEP, that took a programmatic approach with a fixed curricla. One benefit of a programmatic approach is that it can be more readily outsourced to others (i.e., community-based partners) than when it is integrated into teacher roles. Yet if an effective whole-school intervention (such as SEHER) is reduced to its programmatic elements in the absence of connection to its wider intervention ethos, then it risks neglecting the essence of the program and potentially, the mechanisms driving change. Indeed in the SEHER intervention, mediation analyses showed that a nurturing school environment (supportive and engaged relationships with teachers and peers, a sense of belonging, and active participation in school climate) was the mechanism through which lower rates of depressive symptoms, experiences of bullying, and perpetration of violence occurred at follow-up (27). The value of school social–emotional environments and supportive relationships within schools for mental health (the focus of the SEHER interventions) was emphasized in a recent systematic review of longitudinal studies that found that over time, higher levels of school connectedness were associated with lower levels of depressive and anxiety symptoms in secondary school students (7). These findings are also consistent with previous research demonstrating that whole-school elements that change school climate have “flow-on” effects for health (e.g., reducing substance use) that affect later cohorts of students (28). This suggests that non-programmatic elements (e.g., pedagogical aspects, policy requirements, teacher training, and supervision) can lead to wider benefits for health in ways that may well be more efficient than what can be achieved through specific health programs.

This tendency to adopt a programmatic approach may be explained by several factors. Some teachers may not have had the knowledge, skills, and attitudes around whole-school approaches and be insufficiently orientated to the links between health and education or approaches that enhance school climate. Further, it may be that teachers did not feel that they had the capacity or permission (at the school leadership or school policy level) to effect change, whether inside or outside of the classroom. There may also have been the assumption from the external provider that both the activities and ethos of the SEHER intervention would permeate through the school community following the program closure by virtue of being a “whole-school” approach. The health sector, including those involved in research, has traditionally viewed schools as a platform for delivering health promoting interventions through the curriculum (e.g., sexual and reproductive health), programs (e.g., school meals), or health services (e.g., immunization) (29). Even when interventions are framed as multi-level or “whole-school,” they are not typically designed in a way that recognizes that schools are complex, adaptive systems that constantly evolve to the needs and priorities of students and the school community and in response to the input conditions (e.g., resourcing, attitudes) (30). Some teachers in this study appreciated and made efforts to promote the more relational aspects of the intervention (e.g., respectful engagement, active listening, engaging with and responding to individual student concerns). While these elements could very efficiently be provided by teachers, regardless of the continuation or not of the SEHER intervention, most teachers did not perceive these elements as constituting the intervention.

Together, these findings indicate the importance of governance structures for the sustained implementation of programs (13). Investment and leadership from the Department of Education in Bihar was fundamental, and our findings also highlight that the governance role of external providers including researchers was also critical (12). SEHER was originally designed and implemented as a randomised controlled trial of a health intervention within a research context and supported by funding external to the school system. The findings suggest that planning for sustainability within the design of an intervention and in partnership with key stakeholders is important or crucial. Ensuring appropriate handover of governance from the external research provider to the Department of Education appears a critical aspect of gaining Department of Education buy-in, which our findings showed was also important for individual schools. This will include explicit and ongoing training and support for schools, as the programmatic elements of the program will not automatically generalize to other areas of school life, without which the knowledge and skills will disproportionately fall to a small number of motivated teachers and threaten sustainability. Funding to support the non-teacher staff roles (e.g., critical friend, SM) who can champion the program and engage in a distributed model of leadership appears critical. In this study, facilitators outside of the school were important for building the motivation of the SM, equipping them with updated resources, and providing mentorship that connected the SM to others in the school. This is potentially reflected in the results of the original SEHER trial which showed that the intervention was not effective in the teacher as SM arm, but was effective in the SM arm where the SM was able to incorporate the wider elements of a whole-school approach. However, the challenge faced by any robust trial—where it cannot be assumed in advance what the findings will be—is that typically, the research budget is spent on running the trial (31). There is a necessary hiatus between completing the trial and having results available to share with stakeholders who may be responsible for funding the ongoing intervention. In that interregnum, even when there is interest in sustaining the intervention should it be found to be effective, momentum and interest risks being lost by individual schools when staff move on and other priorities emerge. This challenge may remain even if researchers manage to obtain funding to support ongoing implementation.

To shift this perspective, there is a growing appreciation of the wider opportunities for health promotion through whole-school systems approaches, such as WHO and UNESCO’s Global Standards for Health Promoting Schools and Systems (31) and its implementation guidance (3, 32). This approach aims to integrate health promotion into the daily practices or culture of a school, reinforcing knowledge and skills outside the classroom, and engaging school staff, parents, and local communities (i.e., fundamentally appreciating links between health and education). This framework aims to aid the progressive sustainable implementation of health promotion in schools by addressing the governance required by governments and school leaders, including the need for health and education sector collaboration, by engaging students and developing collaborative networks within schools, reviewing and integrating existing programs, building teacher capacity and monitoring and evaluation. The findings of this case study reinforce this approach, especially the importance of authorizing environments and building teacher capacity (e.g., training, and supervision). Additionally, these results imply that funding agencies and researchers need to invest in developing innovative evaluation frameworks to establish implementation science indicators including maintenance, transferability, and sustainability for such interventions within project cycles.

This case study provides the first empirical evidence of how the desire to sustain a whole-school health promotion intervention and the continuation of intervention activities in low-resource setting schools in India depended on individual, school-level, and government-level factors, and the degree of support provided by the external provider of the program after its formal closure. Notwithstanding the importance of understanding a rural Indian context in order to address these issues, the barriers and enablers identified in this study are similar to those found in other studies, including in HICs [e.g., (11, 12, 33)]. It addresses an important gap in implementation research as a dearth of studies have examined the sustainability of whole-school health promotion programs, especially in LMICs. As a result of the ongoing COVID-19 pandemic restrictions, data were obtained from a limited number of schools via convenience sampling. While we included the important stakeholders in a school community, including students, these findings require cautious consideration when transferring to other settings, even if notionally similar. We adopted a multi-stage and iterative process of data interpretation, however, there is scope for observer bias due to the nature of qualitative data, particularly as Sangath staff conducted the interviews. However, participants’ critical comments about Sangath’s role in ongoing implementation suggest that this is unlikely to have been a major concern. Due to limited resources and constraints because of the COVID-19 pandemic, we could not interview representatives from the Department of Education and SEHER intervention staff. Their opinions and positions would have further enriched our findings.

## Conclusion

Many school-based health promotion programs are developed and evaluated by external providers which present challenges to sustainability when initial funding and support ceases. Four key sustainability constructs emerged. Firstly, it is critical to collectively develop an understanding of the educational environment and socio-political context during the intervention co-creation and implementation stages. Effective health programs will not necessarily become embedded within the day-to-day operations of a school simply because they are designed to take a “whole-school” approach. Rather, it is necessary for researchers to plan for program sustainability beyond the initial trial period early in the study design and in consultation with school-level and government-level stakeholders, and potential funders. Arguably, this is particularly important in settings where there is reliance on external providers to deliver interventions. External providers must look beyond the resourcing required to continue specific (especially didactic) components of the program itself and also consider the required governance structures, partnerships, and training. This process should be balanced with the possibility that the intervention should not be sustained if trial results indicate it is ineffective or that the process of sustainability necessitates a substantial deviation from a manualized program. Secondly, it is crucial to invest in the development of committed leadership and motivation among school staff, as this can facilitate the integration of the intervention into school policies and day-to-day functions. Assisting school staff to recognize the importance of student engagement is part of this task. Thirdly, seamless integration of intervention activities into school practices and support from networks both in and outside the school requires investment in translating the program’s philosophy and underpinning principles across the school community throughout the project cycle. Finally, school administration and teachers’ commitment and support, observations of positive impact on students’ behavior and well-being, and confidence in delivering health promotion and their belief in its value may facilitate or prohibit schools from sustaining health interventions. The identification of resources and processes, including those that support convergence between health and education sectors, is required when considering future sustainability (e.g., phased program closure, identifying roles of different partners, decisions regarding resource and funding allocation, realist evaluations). How this is achieved in the context of uncertain benefit of interventions at the time of their design, implementation and evaluation is an important avenue for future research.

## Data availability statement

The raw data supporting the conclusions of this article will be made available by the authors, without undue reservation.

## Ethics statement

The studies involving human participants were reviewed and approved by this study was approved by the Human Ethics Advisory Committee at The University of Melbourne, Australia (Ethics ID 2057035.1). Written informed consent to participate in this study was provided by the participants’ legal guardian/next of kin. Written informed consent was obtained for all adult participants. For participants under the age of 18, participant assent was obtained, using parental opt-out consent due to the low-risk nature of the research.

## Author contributions

SMS, MR, and SS contributed to the study design and implementation, analysis of results, and writing of the manuscript. On the ground, AS collected data, performed transcriptions, and translated documents. The first draft of the manuscript was prepared by SS and MR, and all authors reviewed it. All authors contributed to the article and approved the submitted version.

## Funding

The India School Engagement Grant (2020) at The University of Melbourne supported the primary data collection for this study through a grant to SMS. MR is supported through the Centre of Research Excellence: Driving Global Investment in Adolescent Health funded by the NHMRC (grant no.: APP1171981).

## Conflict of interest

MR and SMS have received consultancy funds from WHO and UNESCO to produce a body of work related to the “Health Promoting Schools” initiative. SS and AS were employed to implement the SEHER project by Sangath, a not-for-profit organization based in Goa, India.

## Publisher’s note

All claims expressed in this article are solely those of the authors and do not necessarily represent those of their affiliated organizations, or those of the publisher, the editors and the reviewers. Any product that may be evaluated in this article, or claim that may be made by its manufacturer, is not guaranteed or endorsed by the publisher.
