# Supplementary material for: What happens when a whole-school health promotion research trial ends? a case study of the SEHER program in India
Source: Front Psychiatry. 2023 Jun 23;14:1112710. doi: 10.3389/fpsyt.2023.1112710 (PMC10326627; doi:10.3389/fpsyt.2023.1112710)
Supplement: Supplementary file 1 [file Data_Sheet_1.docx]

| **Supplementary File 1: SEHER Case study Codebook Framework**   1. **Current status of the program**    1. Continued    2. Discontinued completely    3. Partially continued for some time/being continued 2. **Context**   2.1 Education system (organizational set-up, governance, hierarchy, challenges, etc.)  2.2 School environment (norms, culture, relationships within school, power dynamics, etc.)  2.3 Local setting (culture, norms, practices, etc.)  **3.** **Closure of the program by Sangath**  3.1 Closure process (communication, handover, etc.)  3.2 Opinions about closure  3.3 Reasons for program closure  **4.** **Understanding of SEHER**  4.1 Among teachers  *4.1.1 Description of the program*  *4.1.2 Activities & topics*  *4.1.3 Underlying principles and whole-school activities (involvement and participation of other stakeholders in the program activities; decision-making process, agency, ownership, multi-layer activities for the school, qualities, values)*  4.2 Among students  *4.2.1 Description of the program*  *4.2.2 Activities & topics*  *4.2.3 Underlying principles*  4.3 Among parents  **5.** **Perceived benefits/impact/value of SEHER**  5.1 Teachers  5.2 Students  5.3 Parents  5.4 School environment  *5.4.1 Social-emotional environment (support, ethos of school, feeling of safety, respected, accepted in/belong to school, interactions, relationships, values, beliefs, etc.)*  *5.4.2 Physical environment (building, infrastructure, physical space, access to school, etc.)*  *5.4.3 Other (curriculum, management, policies, etc.)*  **6.** **Decision to continue at school-level**  6.1 Process of decision-making  6.2 Output of the process  6.3 Considered factors  6.4 Reasons for continuation (Intervention characteristics, perceived impact, etc)  6.5 SM/TSM/s role in continuation  6.6 Principal’s role in continuation  6.7 Students’ role in continuation/student engagement  6.8 Role of parents  6.9 Support received/expected from Sangath in decision-making  6.10 Teachers’ role in continuation  6.11 DoE’s role in continuation  **7. Decision to discontinue at school-level**  7.1 Process of decision-making  7.2 Output of the process  7.3 Reasons for discontinuation  7.4 Possible alternatives discussed and output  7.5 SM/TSM/s role in discontinuation  7.6 Principal’s role in discontinuation  7.7 Teachers’ role in discontinuation  7.8 Students’ role in discontinuation  7.9 Support received/expected from Sangath in decision-making  **8. Adjustment in program for continuation**  8.1 Selection of activities for continuation and reasons  8.2 Modifications/adaptations made for continuation and reasons  8.3 Reasons for dropping activities  **9. Current factors in continuation/sustainability (present)**  9.1 Resources (financial, human, materials, time, etc.)  9.2 Cognitive factors (staff training & capacity)  9.3 Structural/social factors (allocation of available funds, motivation to use funds, attitudes, dynamics with school)  9.4 Other challenges  9.5 Ways to address challenges  **10. Support required for continuation**  10.1 Department of Education  *10.1.1 Directives/orders/letters*  *10.1.2 Support (Funds, other resources, capacity building and supervision, meetings/consultation, etc.)*  10.2 Sangath (coordination, networking/liaising, supervision, monitoring, training and capacity building, financial, etc.)  10.3 Other (, incentives, and other things)  **11. Other programs**  11.1 Description (content, structure)  11.2 Topics (List of topics)  11.3 Delivery agents (teachers, role of teachers, understanding of the role, etc.)  11.4 Challenges (Programmatic, structural, human resource, other resources, other)  11.5 Benefits and values  **12. Integration of SEHER with other programs**  12.1 Possibilities & intentions (Content, activities, context, etc.)  12.2 Challenges (Programmatic, structural, human resource, other resources, other)  12.3 Outputs of any efforts  12.4 Perceived benefits  **13. Willingness to continue SEHER (in the future)**  **14. Other suggestions**  **15. COVID-19**  **Free Nodes**  Health and Education  Learning of the programs  Programmatic values  Interphase between health and education |
| --- |
